# Evidence for Lignocellulose-Decomposing Enzymes in the Genome and Transcriptome of the Aquatic Hyphomycete *Clavariopsis aquatica*

**DOI:** 10.3390/jof7100854

**Published:** 2021-10-12

**Authors:** Felix Heeger, Elizabeth C. Bourne, Christian Wurzbacher, Elisabeth Funke, Anna Lipzen, Guifen He, Vivian Ng, Igor V. Grigoriev, Dietmar Schlosser, Michael T. Monaghan

**Affiliations:** 1 Department Ecosystem Research, Leibniz Institute of Freshwater Ecology and Inland Fisheries (IGB), 12587 Berlin, Germany; lizbourne82@gmail.com (E.C.B.); funke@igb-berlin.de (E.F.); monaghan@igb-berlin.de (M.T.M.); 2 Department Materials and Environment, Federal Institute for Material Research and Testing, 12203 Berlin, Germany; 3 Berlin Center for Genomics in Biodiversity Research, 14195 Berlin, Germany; 4 Chair of Urban Water Systems Engineering, Technical University of Munich, 85748 Garching, Germany; c.wurzbacher@tum.de; 5 US Department of Energy Joint Genome Institute, Lawrence Berkeley National Laboratory, Berkeley, CA 94720, USA; alipzen@lbl.gov (A.L.); ghe@lbl.gov (G.H.); vng@lbl.gov (V.N.); ivgrigoriev@lbl.gov (I.V.G.); 6 Department of Plant and Microbial Biology, University of California, Berkeley, CA 94720, USA; 7 Department of Environmental Microbiology, Helmholtz Centre for Environmental Research—UFZ, 04318 Leipzig, Germany; dietmar.schlosser@ufz.de; 8 Institut für Biologie, Freie Universität Berlin, 14195 Berlin, Germany

**Keywords:** aquatic fungi, differential expression, lignocellulose, laccase, RNA-Seq

## Abstract

Fungi are ecologically outstanding decomposers of lignocellulose. Fungal lignocellulose degradation is prominent in saprotrophic Ascomycota and Basidiomycota of the subkingdom Dikarya. Despite ascomycetes dominating the Dikarya inventory of aquatic environments, genome and transcriptome data relating to enzymes involved in lignocellulose decay remain limited to terrestrial representatives of these phyla. We sequenced the genome of an exclusively aquatic ascomycete (the aquatic hyphomycete *Clavariopsis aquatica*), documented the presence of genes for the modification of lignocellulose and its constituents, and compared differential gene expression between *C. aquatica* cultivated on lignocellulosic and sugar-rich substrates. We identified potential peroxidases, laccases, and cytochrome P450 monooxygenases, several of which were differentially expressed when experimentally grown on different substrates. Additionally, we found indications for the regulation of pathways for cellulose and hemicellulose degradation. Our results suggest that *C. aquatica* is able to modify lignin to some extent, detoxify aromatic lignin constituents, or both. Such characteristics would be expected to facilitate the use of carbohydrate components of lignocellulose as carbon and energy sources.

## 1. Introduction

The aquatic hyphomycetes (AQH; Ingoldian fungi) are a polyphyletic group of fungi, most of which belong to the phylum Ascomycota and some of which belong to the Basidiomycota, are major decomposers of lignocellulosic plant litter found submerged in aquatic environments but that most likely originated from the terrestrial environment [1,2]. Whereas it is generally accepted that AQHs can degrade and utilize cellulose and hemicellulose from plant litter, the available knowledge about the enzyme systems involved is astonishingly scarce compared with that gathered from terrestrial fungi [1,3]. Moreover, the potential for AQHs to degrade lignin, the third major component of lignocellulose, and the corresponding candidate enzymes potentially contributing to lignin biotransformation by these exclusively aquatic fungi are even less well understood [1].

Lignin is the most recalcitrant macromolecule among the major lignocellulose constituents and is composed of phenylpropanoid monomer units. The proportions of the three lignocellulose components vary considerably among plant species, with the highest proportion of lignin occurring in wood [4]. Fungal lignin degradation has been studied intensively in terrestrial members of the Basidiomycota and also in terrestrial ascomycetes [4,5,6]. Basidiomycetes causing a white-rot type of lignin degradation (i.e., a substantial mineralization of lignin to CO_2_ and H_2_O) involve wood-rotting and litter-decaying species and employ extracellular lignin-modifying enzymes such as laccases and the class II secretory heme peroxidases (PODs) lignin peroxidase (LiP; EC 1.11.1.14), manganese peroxides (MnP; EC 1.11.1.13), and versatile peroxidase (VP; 1.11.1.16) for lignin breakdown [4,5,6]. By contrast, brown-rot basidiomycetes can only modify lignin to a lesser extent and lack the ligninolytic class II PODs [4,5,6]. Certain terrestrial ascomycetes are known to cause the soft rot of woody tissues, which also involves lignin modification and have been reported to secrete laccase as the only lignin-modifying enzyme [4,7]. According to RedoxiBase (http://peroxibase.toulouse.inra.fr, accessed 13 July 2021), ligninolytic class II PODs are restricted to Basidiomycota. At the same time, comparative studies of the genomes of wood decaying basidiomycetes suggest that common decay-type classifications such as white rot and brown rot oversimplify the full functional diversity of fungi dwelling on lignocellulosic substrates [6,8]. This may also apply to fungal phyla other than Basidiomycota. Beyond genome analysis alone, greater insight into the proteins produced during lignocellulose degradation can be gained from accompanying studies of gene expression (e.g., [9,10]).

*Clavariopsis aquatica* is an exclusively aquatic (freshwater) cosmopolitan ascomycete that colonizes and degrades leaf litter in streams [1,11,12,13]. It has been reported to biotransform environmental surface-water pollutants such as nonylphenol and polycyclic musk fragrances in a cometabolic manner, thereby involving both extracellular laccase and intracellular oxidation reactions that are indicative of the action of cytochrome P450 systems [1,14,15]. In basidiomycetes that play a role in wood and litter decay, both enzyme classes have been implicated in the oxidative breakdown of natural plant-derived compounds such as lignin constituents of lignocellulose and of numerous environmental pollutants of xenobiotic origin [5,16,17,18]. Because of the various and sometimes multiple functions of these protein families, and the frequent occurrence of their members in many different forms, the function of the identified genes cannot easily be inferred from their nucleotide sequence alone. The ability of aquatic ascomycetes such as the AQH *C. aquatica* to effectively degrade plant material and xenobiotics under submerged conditions [1] renders this group of fungi potentially interesting for biotechnological applications. However, the molecular basis of lignocellulose decomposition and breakdown of environmental pollutants by strictly aquatic fungi such as freshwater ascomycete AQHs has not yet been investigated in detail. Corresponding genome studies can further help to fill still existing gaps in the available genomic resources of the Ascomycota [19].

Using *C. aquatica* as a model organism, we sequenced a genome of this exclusively aquatic fungal species for the first time. We then searched for laccase, peroxidase, and cytochrome P450 enzyme systems known to act on lignocellulose components and organic environmental pollutants in other fungi. We also used a replicated incubation assay to examine differential gene expression during growth on substrates with varying lignin content (alder leaves, wheat straw, and malt extract). Common alder (*Alnus glutinosa*) leaves are a typical natural substrate of *C. aquatica* and other aquatic hyphomycetes in rivers and have been widely used as a growth substrate in studies involving aquatic hyphomycetes [1]. Wheat straw is a lignocellulosic substrate not typically found in *C. aquatica* habitats but has a higher cellulose and lignin content than alder leaves [20,21,22,23] and thus might prompt a stronger signal of differential expression of genes related to the metabolism of these polymers. Malt extract is a sugar-based substrate devoid of phenolic and other aromatic constituents and serves as a control for comparison. We examined annotations of differentially expressed genes for over-represented annotation terms in an effort to identify critical pathways that may be involved in fungal carbon cycling under submerged conditions. To our knowledge, this is the first combined genome and gene expression study of an exclusively aquatic fungus.

## 2. Materials and Methods

*Clavariopsis aquatica* De Wild. strain WD(A)-00-01 was made available from the strain collection of the Department of Environmental Microbiology at the Helmholtz Centre for Environmental Research—UFZ (Leipzig, Germany) and was maintained on agar plates containing 1% malt extract (*w*/*v*) and 1.5% agar (pH 5.6–5.8) [14]. Liquid cultivations of *Clavariopsis aquatica* were carried out in 500 mL flasks containing 200 mL of the media described in the following. For cultivation on lignocellulosic substrates, 10 g/L milled alder leaves or wheat straw (particle size ca. 2–4 mm) were autoclaved (121 °C, 20 min) twice and suspended in a nitrogen-limited medium previously described for manganese peroxidase production in *Stropharia rugosoannulata* [24]. This medium contained (per liter) 2.0 g KH_2_PO_4_, 0.5 g MgSO_4_ · 7 H_2_O, 0.1 g CaCl_2_, 0.22 g diammonium tartrate, 10 mL mineral stock solution (composition per liter: 3.0 g MgSO_4_ · 7 H_2_O, 1.5 g nitrilotriacetate, 1.0 g NaCl, 1.7 g MnSO_4_ · H_2_O, 181.2 mg CoSO_4_ · 7 H_2_O, 178 mg CaCl_2_ · 2 H_2_O, 100 mg FeSO_4_ · 7 H_2_O, 100 mg ZnSO_4_, 18.4 mg AlK(SO_4_)_2_ · 12 H_2_O, 12 mg NaMoO_4_ · 2 H_2_O, 10 mg CuSO_4_ · 5 H_2_O, and 10 mg H_3_BO_3_) and 1.5 mL vitamin stock solution (composition per liter: 10 mg pyridoxine hydrochloride, 5 mg 4-aminobenzoic acid, 5 mg calcium DL-pantothenate, 5 mg liponic acid, 5 mg nicotinic acid, 5 mg riboflavin, 5 mg thiamine hydrochloride, 2 mg biotin, 2 mg folic acid, and 0.1 mg cyanocobolamin). Control cultures were grown on liquid malt extract medium (1% malt extract, *w*/*v*; pH 5.6–5.8) [25]. The flasks were inoculated with 5 mL of a mycelial suspension of *C. aquatica* prepared in sterile water [14]. Cultures were agitated at 120 rpm and incubated at 14 °C in the dark. Flasks were harvested after 7 days (trophophase/exponential growth phase) and 20 days (stationary growth phase) of cultivation [14,25]. Liquid media were removed by stepwise centrifugation of the content of a flask in sterile 50 mL conical tubes at 7197 g and 4 °C for 10 min (Eppendorf centrifuge 5430R, rotor FA-35-6-30; Eppendorf, Hamburg, Germany). After discarding respective supernatants, biomass pellets obtained from one cultivation flask were combined in one sterile 50 mL conical tube, shock frozen in liquid nitrogen, and kept frozen at −80 °C until RNA extraction.

Fungal growth phases and related starvation conditions are well known to play important roles in the regulation of enzymes involved in lignocellulose degradation [25,26]. Liquid culturing with milled plant material allows for clear temporal differentiation between exponential and stationary growth phases under laboratory conditions and was applied in this study. To mimic more natural conditions with respect to the potential co-existence of different fungal growth phases at the same time point, as would be expected during fungal colonization and growth on suspended and bulky solid plant material [1,2], additional cultures were grown on comparatively more inhomogeneous solid substrates (i.e., not suspended in solution/water).

For cultivation on solid substrate (wheat straw or alder leaves), 100 mL flasks were supplemented with 2 g (dry mass) of milled substrate (about 2–4 mm particle size) and 8 mL of tap water and autoclaved (121 °C, 20 min) twice. The flasks were inoculated with six mycelia-containing agar plugs, derived from the edge of *C. aquatica* colonies on malt extract agar plates [14], and incubated without agitation at 14 °C in the dark. Flasks were harvested after 26 days of cultivation, and solid substrates were shock frozen in liquid nitrogen and kept frozen at −80 °C until RNA extraction.

Overall, there were three conditions (liquid culture during stationary growth, liquid culture during exponential growth, and solid-state culture) for each of the natural substrates (wheat straw and alder leaves) and two conditions (liquid culture during stationary and exponential growth phases) for malt extract. All sampling was performed in triplicate, which led to 24 samples in total. Sterile conditions were ensured throughout sampling.

*C. aquatica* WD(A)-00-1 mycelium was extracted from agar plates and subjected to DNA extraction using the FastDNA Spin Kit for Soil (MP Biomedicals, Irvine, CA, USA) following the manufacturer’s instructions. Whole-genome shotgun reads of the *C. aquatica* genomic DNA were generated using a NexteraXT library preparation kit (Illumina, San Diego, CA, USA) following the manufacturer’s protocol and sequenced on a MiSeq (paired-end, 300 bp) instrument with the v3 chemistry (Illumina) after library verification with a nano kit (Illumina).

Frozen material from each sample was ground to a fine powder using an RNase-cleaned and precooled pestle and mortar and liquid nitrogen, with a small spatula of zirconium beads (Biospec, Bartlesville, OK, USA) added for additional friction. RNA extraction followed Bourne et al. [27] and Johnson et al. [28] using a CTAB-based extraction. In brief, for each sample, ca. 500 mg of ground, frozen tissue was added to 1.4 mL preheated (65 °C) CTAB buffer, vortexed until thoroughly mixed, incubated at 65 °C for 10–15 min, and centrifuged at 13,000× *g* for 3 min. The supernatant was transferred to a new 2 mL tube for two rounds of chloroform:isoamyl (24:1) extraction, a single phenol-chloroform extraction (5:1, pH 4.5), and a final chloroform:isoamyl (24:1) extraction. Following centrifugation, the upper phase was transferred to a new 2 mL tube. Purification was performed using the RNeasy Mini Kit (Qiagen, Hilden Germany) with on-column DNA digestion (RNase-free DNase set, Qiagen), following the manufacturer’s guidelines. RNA was eluted by adding 30 µL of elution buffer directly to the membrane and spinning at 13,000× *g* for 1 min.

Total RNA was quantified using the QuantiFlour RNA system (Promega, Madison, WI, USA). The presence of DNA was checked using the QuantiFlour DNA system (Promega), and samples with remaining DNA underwent an additional postextraction DNase treatment using the TURBO DNA-free kit (Invitrogen, Thermo Fisher Scientific, Waltham, MA, USA) following the manufacturer’s guidelines. Integrity of the RNA was assessed with the Agilent RNA 6000 Nano Kit and Agilent 2100 Bioanalyzer (Agilent Technologies, Santa Clara, CA, USA) following manufacturer’s guidelines. The RNA integrity (RIN) value was determined as a proxy of the overall quality of the RNA sample, with a value greater than 6 considered suitable for further analysis. We also assessed the quality of the overall bioanalyzer trace by eye. Multiple extractions were performed for each sample and pooled to obtain sufficient RNA quantity for sequencing. Samples with sufficient quality were sent on dry ice for library preparation and sequencing (see below). RNA of sufficient quantity and quality could not be obtained from all samples. The sampling points for stationary growth in liquid culture and on the solid substrate for alder leaves had to be excluded, as well as for stationary growth on malt extract. For exponential growth in liquid culture for alder leaves, only two of the replicates produced suitable quantities of RNA. In total, 14 samples were used for library preparation.

RNA library preparation and sequencing was performed at the DOE Joint Genome Institute in Walnut Creek, CA, USA. Stranded cDNA libraries were generated using the Illumina Truseq Stranded RNA LT kit. mRNA was purified from 1 µg of total RNA using magnetic beads containing poly-T oligos. mRNA was fragmented and reverse-transcribed using random hexamers and SSII (Invitrogen), followed by second-strand synthesis. The fragmented cDNA underwent end-repair, A-tailing, adapter ligation, and 8 cycles of PCR. qPCR was used to determine the concentration of the libraries. Libraries were sequenced on the Illumina Hiseq (single-end, 100 bp).

Reads were digitally normalized with khmer (version 0.7.1, [29]) to remove read errors and reduce computation times, as follows: In the first step, reads were normalized to a coverage of 20 [30]. After removal of low-abundance kmers [31], another round of normalization to a nonredundant coverage of 5 was applied (Supplementary Materials S1) and only read pairs with both reads remaining after normalization were used for assembly. Assembly was performed with velvet (version 1.2.10, [32]) and run with different kmer lengths k (see Supplementary Materials S1 for details). Finally, k = 27 was chosen because it resulted in the highest N50 score. To estimate genome completeness, we ran BUSCO (version 3.0.2, [33]) with the pezizomycotina reference set of single-copy genes. The clean command of the funannotate pipeline (version 1.2.0, [34]) was used to remove contigs shorter than 500 bp as well as redundant contigs.

Reads obtained from RNA-Seq were de novo assembled into putative transcripts with Trinity (version 2.5.1, [35]). Trinity was configured to use trimmomatic for trimming and to perform digital normalization (see Supplementary Materials S1 for further details). Normalized RNA-Seq reads produced by Trinity were mapped to the genome contigs with Star (version 2.5.3a, [36]), using default parameters. The mapped reads were used to generate a genome-guided assembly with Trinity (see Supplementary Materials S1 further details). The PASA pipeline (version 2.2.0, [37]) was used to combine de novo and genome-guided assembled transcripts into a single gff file as evidence for annotation (Supplementary Materials S1). The resulting gff file together with genome-guided assembled transcripts and mapped reads were used as input for the predict command of the funannotate pipeline. The update command of funannotate was then used to add UTR annotations. The predicted protein sequences were used as input for Interproscan (version 5.27, [38]) to generate Interpro [39] as well as gene ontology (GO) [40,41] annotations. The annotate command of the funannotate pipeline was used to combine Interproscan results with CAZy [42] annotations from dbCAN (version 6.0, [43]).

In addition to the annotations from the funannotate pipeline (above), proteins were assigned as either secreted or not secreted using signalP (version 4.1, [44]) and to KEGG (Kyoto Encyclopedia of Genes and Genomes) [45] orthology groups with the BlastKOALA web service [46].

Besides general genome annotation, we specifically searched for gene families known to be involved in lignin degradation. We performed a blast search against our newly assembled *C. aquatica* genome to check for five previously described, partially sequenced laccase genes [25]. In addition, we identified multicopper oxidases (MCO) by assignment to the CAZy family AA1. MCOs were further classified using a blast search of the Laccase and Multicopper Oxidase Engineering Database (version 6.4, [47]). We identified possibly relevant peroxidases by annotation with the Interpro family IPR001621 (Fungal ligninase) and verified the resulting proteins by annotation with the Peroxiscan [48] web service (accessed on 15 May 2018) of PeroxiBase [49]. Proteins potentially belonging to the cytochrome P450 family were identified by annotation with the Interpro family IPR001128 (Cytochrome P450).

Read counts per gene were generated with RSEM (version 1.3, [50]) using default parameters from mapped RNA-Seq reads (see above). All of the following analyses were implemented as a snakemake (version 3.5.4, [51]) workflow that can be found at www.github.com/f-heeger/caquatica_expression. RSEM output files were combined into a single read count matrix with the merge_RSEM_output_to_matrix.pl script from Trinity. Differential gene expression between different samples was modeled with the DESeq2 (version 1.10.1, [52]) R package. Different DESeq2 models were created for different comparisons (wheat straw versus malt extract, alder leaf versus malt extract, etc.). Differential expression statistics were generated with the *results* function of DESeq2 using the *p* value cutoff of 0.05 (see below) as input (*alpha* parameter) for the independent filtering. From the resulting list, genes with an adjusted *p* value (FDR) < 0.05 and an absolute log_2_ fold change >1 (>200% over- or under-expressed) were considered to be differentially expressed.

To interpret the result, genes were grouped into sets according to their functional annotations. We defined gene sets using three different annotations: (1) all genes annotated with one GO term, (2) all genes assigned to one CAZy family, and (3) all genes assigned to one KEGG pathway based on assignment to KEGG orthology groups (see above). Regulation of each gene set in a given comparison (e.g., wheat straw versus alder) was estimated with multiple gene set activation (MGSA) analysis. MGSA uses a Bayesian network approach to predict a probability of activation for sets of genes for each comparison based on differentially expressed genes [53,54]. We used the R package *mgsa* [54] to perform the analysis. The activation probability cutoff, above which a gene set is considered to be “activated”, is ultimately arbitrary. The authors of the method suggest to use 0.5 [53], reasoning that this means that the gene set is “more likely to be on than to be off”. We chose a slightly more conservative cutoff of 0.6 for gene sets to be considered activated. We note that activation is a statistical term, here indicating that differential expression of genes in these sets can be best explained by some form of regulation of these sets, given the Bayesian model underlying the MGSA analysis.

## 3. Results

### 3.1. Genome Assembly and Annotation

We obtained 7.31 million read pairs (2 × 300 bp), which were deposited in the NCBI Sequence Read Archive under the accession PRJNA610219. They were reduced to 1.09 million reads by digital normalization and assembled into 2650 nonredundant contigs (longer than 500 bp) with a N50 score of 30,079 bp and a total length of 34.18 Mb. These included complete single copies of 94.8% of the single-copy genes expected according to the BUSCO reference set, indicating good completeness of our assembly. The genome assembly was deposited at DDBJ/ENA/GenBank under the accession JAAZQE000000000 and can be found in Mycocosm [55] under mycocosm.jgi.doe.gov/Claaq1. A total of 12,100 proteins were predicted by the funnanotate pipeline, of which 6128 (50.64%) were annotated with at least one GO term, 2322 (19.19%) were assigned to at least one KEGG pathway, and 572 (4.73%) to at least one CAZy family. There were 5724 (47.31%) proteins that did not receive any annotation from these databases.

All five of the laccase gene sequences previously described from *Clavariopsis aquatica* [25] were present in our genome, with nucleotide identity >98%. Based on annotation with CAZy auxiliary activity family AA1, we identified all five known laccases and eight additional multicopper oxidases. They all exhibited a high degree of similarity (53–100% pairwise amino acid identity) for conserved sites of laccase genes [56] in a multiple alignment (data not shown). When compared to the Laccase and Multicopper Oxidase Engineering Database with blast, only one of the previously described laccases (lcc2) was classified as belonging to the “Basidomycete Laccase” superfamily in the database. The other four were assigned to the superfamily “Ascomycete MCO”. Of the newly identified potential multicopper oxidases, five were assigned to “Ascomycete MCO” as well, while the other three were classified as “Fungal Ferroxidase” and are not considered further.

Based on annotation with the Interpro family IPR001621, we identified five peroxidases. Annotation with Perociscan, verified all as belonging to the class II peroxidase superfamily in PeroxiBase and further assigned them to the “Asco Class II” family. No dye-decolorizing (DyP-type) peroxidases [4] could be identified by searching InterPro annotations for the ID IPR006314.

A total of 137 proteins were identified as belonging to the cytochrome P450 superfamily by annotation with the Interpro family IPR001128.

### 3.2. RNA-Sequencing and Differential Expression

We obtained 317.18 million RNA-Seq single end reads (100 bp) in total, with >14 million reads for each sample (see Table 1). Reads were deposited in the NCBI Sequence Read Archive under the accessions PRJNA440444–PRJNA440457. Most (75.27%, SD 1.33%) of the reads from each sample could be mapped to our newly assembled *C. aquatica* genome with RSEM. Two of the samples (both liquid culture, exponential phase on wheat straw) had considerably more reads than the rest (50.50 and 57.49 million). Subsampling to 17 million reads (rounded mean number of reads in the other samples) and rerunning RSEM mapping and differential expression analysis with DESeq2 showed only minor differences (97.55% genes with the same expression status). Because of this result and considering that read count per sample is accounted for in the DESeq2 model, we used all reads without subsampling for further analyses. We modeled differential expression between recalcitrant and nutrient-rich media (wheat straw versus malt extract and alder versus malt extract), between the two growth phases on wheat straw (stationary versus exponential), and among methods of culture (solid culture versus exponential growth in liquid culture and solid culture versus stationary growth in liquid culture).

Gene expression showed strong differences between solid and liquid cultures, with the most differentially expressed genes being between growth in solid culture compared to stationary growth in liquid culture and the second-most differentially expressed genes between solid growth and exponential growth in liquid culture. The difference between stationary and exponential growth was weaker by comparison (see Table 2). Growth on both plant matter substrates induced similar changes in the metabolism compared to malt extract, indicated by a significant (Fisher’s exact test, *p* < 10–192) overlap between differentially expressed genes.

**Figure 1:**
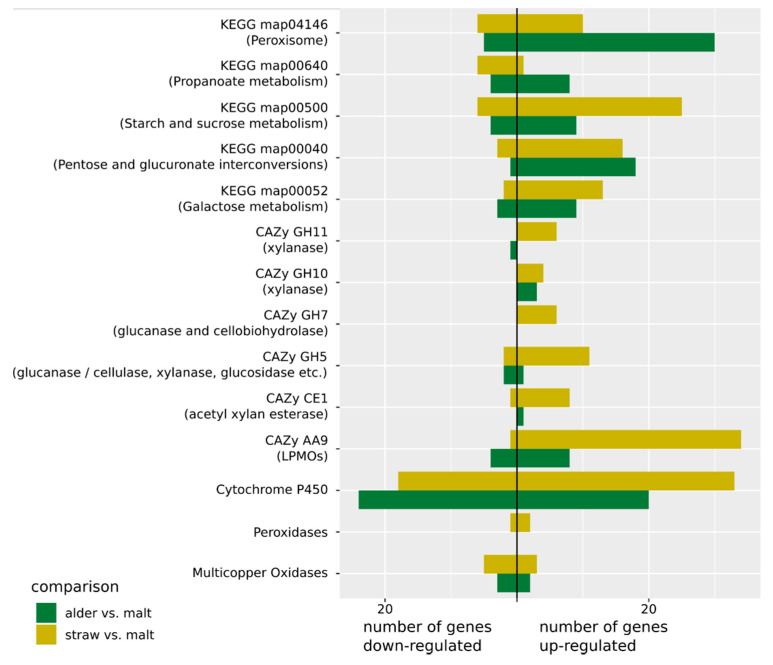
Number of up- and down-regulated genes in different gene groups for the comparison between wheat straw versus malt extract (yellow) and between alder leaves versus malt extract (green).

The five known laccase genes, as well as the eight newly identified laccase-like genes, showed no consistent pattern of up- or down-regulation in the exponential growth phase in liquid culture when comparing alder and wheat straw (Figure 1). Of the six identified putative Class II peroxidases, two were upregulated on wheat straw and one was downregulated on wheat straw. For growth on alder, no significant differential expression was observed for the putative peroxidases (Figure 1). Of the 137 possible cytochrome P450 proteins, 33 were up- and 18 down-regulated in wheat straw, and 20 were up- and 24 down-regulated on alder (Figure 1).

### 3.3. Gene and Gene Set Activation

We found multiple activated GO terms, KEGG pathways, and CAZy families for all comparisons (Supplementary Tables S3–S5). The only exception was the comparison between the exponential growth phase on alder leaves and on malt extract, where no active CAZy family was identified. We concentrate here on the differential expression between exponential growth on wheat straw and on malt extract, and between exponential growth on alder leaves and on malt extract, because they are the most relevant when investigating biomass degradation (Figure 1).

From the six CAZy families that were predicted by MGSA to be regulated for growth on wheat straw (Supplementary Table S2A), three (CE1, GH10, and GH11) were linked to xylane and thus hemicellulose degradation [57], and two (GH7 and GH5_5) were linked to glucan and cellulose degradation. The CAZy family predicted to be regulated with the most genes was AA9, which contains lytic polysaccharide mono-oxygenases (LPMOs) acting among others on cellulose to prepare it for further enzymatic degradation and has been shown to degrade hemicellulose as well (Agger et al., 2014). Investigation of the expression of the genes assigned to these groups in the *C. aquatica* genome showed that for growth on wheat straw they were almost all strongly upregulated, while for growth on alder in most cases (except for GH10) there was no or only weak upregulation. For each of the families CE1 and GH7, there was one of the assigned genes that was not upregulated. This was also the only gene in these families predicted (by signalP) to be not secreted.

The nonsignificant (Fisher’s exact test, *p* = 0.0512) overlap between predicted activation of KEGG pathways (Supplementary Table S2B) for growth on alder and wheat straw contained the two pathways ko00040 (Pentose and glucuronate interconversions) and ko00052 (Galactose metabolism). The upregulation of the pentose and glucuronate interconversions pathway was mostly caused by the upregulation of the genes on the path from pectin to glycerol and regulation of some genes involved in conversion of xylose to ribulose. The upregulated enzymes in the galactose metabolism catalyze conversion of galactose into glucose. The pathway ko00500 (starch and sucrose metabolism) was only predicted to be regulated for growth on wheat straw by the MGSA analysis. Most of the regulated genes are involved in cellulose degradation into glucose, but there is also down regulation of conversion of maltose into glucose. Although this pathway was not predicted to be regulated for growth on alder, many of the genes showed differential expression for that comparison as well. Two interesting pathways predicted to be activated for growth on alder, but not on wheat straw, were ko04146 (Peroxisome) and ko00640 (Propanoate metabolism). In ko04146, in addition to multiple genes that are important for structure and function of the peroxisome, genes involved in the β-oxidation in the peroxisome were upregulated. In ko00640, genes for the degradation of propanoate through the β-oxidation into Acetyl-CoA were upregulated.

The activated GO terms (Supplementary Table S2C) were mostly connected to metabolism but were not specific enough to lead to any further conclusions. GO terms predicted to be regulated in the comparison between growth on wheat straw versus growth on malt extract had a significant overlap (Fisher’s exact test, *p* < 10–15) with GO terms predicted to be regulated for the comparison between growth on alder leaves versus growth on malt extract.

## 4. Discussion

In this study, we were aiming to identify genes, in an exclusively aquatic fungus, that encode oxidative enzyme systems that potentially contribute to the breakdown of lignocellulose and organic environmental pollutants. We found ten possible laccases in the genome of the aquatic ascomycete *Clavariopsis aquatica*, of which only five had previously been identified [25]. The fact that one of them was assigned to the “Basidomycete Laccase” superfamily was somewhat surprising, given that *C. aquatica* is a member of the Ascomycota; however, further investigation revealed that 196 sequences from a total of seven classes of the Ascomycota (including filamentous ascomycetes, e.g., *Aspergillus oryzae* and *Trichoderma reesei*, and also yeasts such as *Yarrowia lipolytica*) make up a notable proportion (9.13%) of the “Basidomycete Laccase” group in the database, and thus the name of the superfamily is probably just historical. The ten possible laccases exhibited both up- and down-regulation on alder and wheat straw in our differential expression experiment. This result builds upon an earlier study [25] which observed differently regulated laccase genes in *C. aquatica* in response to metals and to xenobiotics and lignocellulose breakdown products and also observed differences among growth stages. The difference we observed between alder and wheat straw may indicate that different *C. aquatica* laccases act on these substrates. The results could also be explained by different laccases being involved at different stages of *C. aquatica* growth and substrate decomposition stages and our cultures on wheat straw and alder being in different growth stages during our experiment. This second explanation seems less likely, because samples were taken at the same time and culture conditions were closely controlled. The multiplicity of laccase genes and their differential regulation is well known from other asco- and basidio-mycete species and has repeatedly been attributed to the possibility of multiple laccase functions within one organism [58,59,60].

We identified five potential peroxidases from the *C. aquatica* genome that were assigned to class II of the nonanimal peroxidase superfamily by Peroxiscan. This class also contains peroxidases known to be involved in lignin degradation in Basidiomycota such as LiP, MnP, and VP [4,5,6]. Other fungal heme peroxidases such as DyP-type peroxidases were also implicated in lignin degradation, although a possibly related function has not yet been finally clarified [61]. The peroxidases identified from the *C. aquatica* genome, however, are more similar to the Ascomycota class II family [62]. Two of these putative peroxidases were upregulated on wheat straw, but not on alder, and to our knowledge, the expression of active peroxidase enzymes has not yet been reported for *C. aquatica*. Their activation on the presumably more lignin-rich wheat straw (compared to alder leaves; see below) could indicate that they are involved in the biotransformation of phenolic lignin constituents, thus possibly contributing to the detoxification of such compounds. The enzymes of the Ascomycota Class II family do not show the specific amino acids to form the active site for lignin degradation with the same mechanisms as MnP, LiP, and VP in Basidiomycota, and their function is not yet understood. Their occurrence in mostly saprotrophic fungi has led to speculation that they might be involved in cell wall penetration and not in lignin decomposition [62].

The third group of enzymes that we investigated belongs to the cytochrome P450 superfamily. Because the classification of these enzymes could not be further specified, it is not clear which of the more than 100 enzymes from this superfamily we found. Potentially, these could act on aromatic structures stemming from lignin or other plant constituents or on aliphatic compounds [17] such as waxes of the leaf cuticle. The observed upregulation of some of these putative cytochrome P450 monooxygenases on wheat straw (n = 33) and alder (n = 20) lends support to this idea.

We only detected clear activation of CAZy families during the exponential growth phase on wheat straw. The activated families all have cellulose- and hemicellulose-degrading activity, as expected on this substrate. The two classical glycoside hydrolase families (GH7 and GH11) that showed the highest activation probabilities have previously been reported to be induced by growth on wheat straw in other fungi [63]. Besides two other glycoside hydrolase families (GH10 and GH5), which are described as acting on cellulose and hemicellulose main chain bonds, we also found upregulation of members of the CE1 family that contains acetyl xylan esterases and of the AA9 family that contains lytic polysaccharide mono-oxygenases (LPMOs). Xlyan esterases cleave hemicellulose side chains, while LPMOs act on cellulose and hemicellulose and have been observed to boost the conversion of lignocellulose via the oxidation of crystalline polysaccharide chains by reactive copper–superoxide complexes [64,65]. The AA9 is a large family and, in our case, 34 genes that were upregulated on wheat straw have been assigned to it. Most of the targets and the specific functions of the variety of LPMOs are not yet clarified [64], but it has been shown that they cleave cellulose and hemicellulose components [66]. Most of the upregulated proteins in the abovementioned CAZy families are predicted to be secreted. Together, this indicates that *C. aquatica* performs extracellular degradation of cellulose and hemicellulose when grown on wheat straw. On alder leaves, none of the CAZy families were predicted as active, and very few of the genes in them were differentially expressed compared to growth on malt extract.

In contrast to this difference, the overall differentially expressed genes on wheat straw and alder showed significant overlap, and two of the KEGG pathways were regulated on both. The genes in the two common KEGG pathways (ko00040 and ko00052) encode enzymes involved in xylose and galactose degradation, and many of the genes for degradation of cellulose into glucose (ko00500) were upregulated on both substrates as well (although many more were upregulated on wheat straw). For growth on wheat straw, this shows a clear process of extracellular cleavage of cellulose and hemicellulose followed by utilization of the monomers as carbon sources.

We identified the upregulation of enzymes involved in the later stages of lignocellulose degradation (e.g., xylose and galactose degradation) on both wheat straw and alder leaves, although we only detected the upregulation of genes for the initial polymer decomposition (e.g., glycoside hydrolases, acetyl xylan esterases, and LPMOs) when *C. aquatica* was grown on wheat straw. One possible explanation is the different composition of the two substrates. Wheat straw contains more cellulose (~40%) and lignin (9–22%) [20,21] than alder leaves (5–15% and 6–20%, respectively) [22,23]. It is possible that this leads to a lower expression of cell wall degrading enzymes when *C. aquatica* is grown on alder leaves. In addition, it may be that alder leaves contain carbon sources other than polysaccharides and that these may be utilized by *C. aquatica*, which tends to grow inside the leaf matrix. Propanoate metabolism (ko00640) was predicted to be activated for growth on alder (Supplementary Table S2B), and enzymes that were upregulated in this pathway in *C. aquatica* have been found to be involved in propanoate degradation via the β-oxidation pathway in other fungi [67]. Potentially, propanoate could be produced from wax-related fatty acids in the alder leaves (for example from those of cutin and suberin not present in wheat straw) or from aliphatic side chains of plant sterols.

## 5. Conclusions

Gene expression of *C. aquatica* on both lignocellulose containing materials showed indication of cellulose and hemicellulose degradation. In particular the enzymes for extracellular depolymerization were more clearly upregulated on the more cellulose rich wheat straw. Multiple laccases, peroxidases, and putative cytochrome P450 monooxygenases were identified in the genome of *C. aquatica*. The expression of several of them was increased on the lignocellulose containing substrates. This observation strongly suggests that *C. aquatica* is able to modify lignin to some extent, perhaps in order to facilitate the utilization of polysaccharide components of lignocellulose as carbon and energy sources. The ecological importance of this process is well established, as is the means by which some of the most persistent and recalcitrant organic matter in aquatic systems is returned to the aquatic food web [1,3,68,69]. Our results shed further light on the potential ecological role of *C. aquatica* and the genomic basis of this capability. Considering the known substrate promiscuity of the aforementioned enzymes, it further emphasizes a role of *C. aquatica* in the breakdown of xenobiotic environmental pollutants when dwelling in its natural riverine habitat.

## Figures and Tables

**Table 1:** *C. aquatica* samples grown under different conditions.

Sample Code	Culture	Medium	Growth Phase	Number of Reads
A3	liquid	wheat straw	exponential	50,666,214
A4	liquid	wheat straw	exponential	57,490,827
A6	liquid	wheat straw	stationary	16,839,738
A7	liquid	wheat straw	stationary	17,367,382
A9	liquid	wheat straw	stationary	17,584,532
B1	liquid	alder leaves	exponential	17,922,153
B3	liquid	alder leaves	exponential	16,700,439
B5	liquid	alder leaves	exponential	14,857,484
D1	liquid	malt extract	exponential	17,796,408
D3	liquid	malt extract	exponential	17,863,281
D4	liquid	malt extract	exponential	19,469,408
E2	solid	wheat straw	NA *	16,859,013
E3	solid	wheat straw	NA *	16,904,004
E4	solid	wheat straw	NA *	18,861,228

* Growth phases appear simultaneously in solid culture and were not separated.

**Table 2:** Number of up- and down-regulated genes for different comparisons.

Comparison		Differential Expression	
Condition 1	Condition 2	Upregulated	Downregulated
exponential growth on wheat straw in liquid culture	exponential growth on malt extract in liquid culture	1430	1570
exponential growth on alder leaves in liquid culture	exponential growth on malt extract in liquid culture	1033	1462
exponential growth on wheat straw in liquid culture	stationary growth on wheat straw in liquid culture	1380	883
growth on wheat straw in solid culture	exponential growth on wheat straw in liquid culture	2731	2328
growth on wheat straw in solid culture	stationary growth on wheat straw in liquid culture	2683	2478

## Data Availability

Whole-genome shotgun sequencing data and RNA-Seq read data were deposited in the NCBI Sequence Read Archive under the accession PRJNA610219 and PRJNA440444–PRJNA440457. The genome assembly was deposited at DDBJ/ENA/GenBank under the accession JAAZQE000000000 and can be found in Mycocosm under mycocosm.jgi.doe.gov/Claaq1.

## Supplementary Materials

The following are available online at https://www.mdpi.com/article/10.3390/jof7100854/s1, Supplementary Methods: Assembly Commands, Table S2: Difference in gene set activation during exponential growth in liquid culture on different carbon sources, Table S3: Differences in CAZy-Family activation, Table S4: Differences in KEGG pathways activation, Table S5: Differences activation of gene sets grouped by GO terms.
