# Supplementary material for: Evidence for Lignocellulose-Decomposing Enzymes in the Genome and Transcriptome of the Aquatic Hyphomycete Clavariopsis aquatica
Source: J Fungi (Basel). 2021 Oct 12;7(10):854. doi: 10.3390/jof7100854 (PMC8537685; doi:10.3390/jof7100854)
Supplement: Supplementary file 1 [file jof-07-00854-s001.zip › jof-1382324-supplementary.pdf]

Supplemental Info S1: Commands

Digital normalization was done with the khmer package with the following steps and parameters:

1. interleave-reads.py
2. normalize-by-median.py -C 20 -k 20 -N 4 -x 2.5e8
3. filter-abund.py clavariopsis.kh
4. normalize-by-median.py -C 5 -k 20 -N 4 -x 1e8
5. extract-paired-reads.py

Genome assembly was done was done with velvet with the digitally normalized reads with the following commands:

1. velveth -fastq.gz -shortPaired
2. velvetg -ins_length 900 -exp_cov 140 -cov_cutoff 50

***De Novo* transcriptome assembly** was done with Trinity with the following command:
Trinity --seqType fq --max_memory 100G --single $INDATA --CPU 12 --trimmomatic --normalize_reads --SS_lib_type R

Genome guided transcriptome assembly was done with Trinity with the following command:
Trinity --genome_guided_bam $INDATA --genome_guided_max_intron 1000 --max_memory 30G --CPU 12

The **PASA pipeline** was run with the following commands:

1. cat Trinity.fasta Trinity-GG.fasta > ${TRANSCRIPTS}
2. accession_extractor.pl < Trinity.fasta > tdn.accs

3. Launch_PASA_pipeline.pl -c alignAssembly.conf -C -R -g ${GENOME} -t ${TRANSCRIPTS} --ALIGNERS blat,gmap --TDN tdn.accs --transcribed_is_aligned_orient --MAX_INTRON_LENGTH 3000
4. build_comprehensive_transcriptome.dbi -c alignAssembly.conf -t ${TRANSCRIPTS} --min_per_ID 95 --min_per_aligned 30

Supplemental Table S1

Supplemental Table S1A: CAZy families predicted to be active by MGSA analysis. For comparison growth on straw compared to growth on malt extract

condition	CAZy Family	activity	genes with this annotation in the genome	differentially expressed genes with this annotation	activation probability
straw-malt	AA9	AA9 (formerly GH61) proteins are copper-dependent lytic polysaccharide monooxygenases (LPMOs); cleavage of cellulose chains with oxidation of various carbons (C-1, C-4 and C-6) has been reported several times in the literature;	49	35	1
straw-malt	CE1	acetyl xylan esterase; cinnamoyl esterase; feruloyl esterase; carboxylesterase; S-formylglutathione hydrolase; diacylglycerol O-acyltransferase; trehalose 6-O-mycolyltransferase	10	9	0.9882
straw-malt	GH11	endo-β-1,4-xylanase; endo-β-1,3-xylanase	6	6	0.96
straw-malt	GH7	endo-β-1,4-glucanase; reducing end-acting cellobiohydrolase; chitosanase; endo-β-1,3-1,4-glucanase	7	6	0.877
straw-malt	GH10	endo-1,4-β-xylanase; endo-1,3-β-xylanase; tomatinase; xylan endotransglycosylase	4	4	0.7582
straw-malt	GH5_5	endo-β-1,4-glucanase / cellulase; endo-β-1,4-xylanase; β-glucosidase; β-mannosidase; β-glucosylceramidase; glucan β-1,3-glucosidase; licheninase; exo-β-1,4-glucanase / cellodextrinase; glucan endo-1,6-β-glucosidase; mannan endo-β-1,4-mannosidase; cellulose β-1,4-cellobiosidase; steryl β-glucosidase; endoglycoceramidase; chitosanase; β-primeverosidase; xyloglucan-specific endo-β-1,4-glucanase; endo-β-1,6-galactanase; hesperidin 6-O-α-L-rhamnosyl-β-glucosidase; β-1,3-mannanase; arabinoxylan-specific endo-β-1,4-xylanase; mannan transglycosylase	5	5	0.6948

Supplemental Table S1B: KEGG pathways predicted to be active by MGSA analysis. For comparison growth on straw compared to growth

on malt extract, and growth on alder compared to malt extract

condition	KEGG pathway ID	KEGG pathway name	genes with this annotation in the genome	differentially expressed genes with this annotation	activation probability
alder-malt	ko00040	Pentose and glucuronate interconversions	35	19	1
alder-malt	ko01120	Microbial metabolism in diverse environments	246	92	1
alder-malt	ko04146	Peroxisome	54	35	1
alder-malt	ko00280	Valine, leucine and isoleucine degradation	49	26	1
alder-malt	ko00640	Propanoate metabolism	28	12	0.9992
alder-malt	ko00460	Cyanoamino acid metabolism	26	13	0.9868
alder-malt	ko00906	Carotenoid biosynthesis	4	4	0.9804
alder-malt	ko04978	Mineral absorption	7	4	0.9438
alder-malt	ko00052	Galactose metabolism	30	12	0.7968
alder-malt	ko04920	Adipocytokine signaling pathway	9	4	0.6656
alder-malt	ko04260	Cardiac muscle contraction	12	4	0.6062
straw-malt	ko00500	Starch and sucrose metabolism	65	31	1
straw-malt	ko00040	Pentose and glucuronate interconversions	35	19	1
straw-malt	ko03008	Ribosome biogenesis in eukaryotes	64	23	1
straw-malt	ko00330	Arginine and proline metabolism	38	18	0.9978
straw-malt	ko00520	Amino sugar and nucleotide sugar metabolism	49	17	0.967
straw-malt	ko00980	Metabolism of xenobiotics by cytochrome P450	28	14	0.9512
straw-malt	ko00630	Glyoxylate and dicarboxylate metabolism	40	21	0.9136
straw-malt	ko00920	Sulfur metabolism	17	8	0.9056
straw-malt	ko00052	Galactose metabolism	30	15	0.897
straw-malt	ko00350	Tyrosine metabolism	50	20	0.838
straw-malt	ko00770	Pantothenate and CoA biosynthesis	23	11	0.7994
straw-malt	ko00910	Nitrogen metabolism	21	9	0.6518
straw-malt	ko01220	Degradation of aromatic compounds	32	17	0.6144

Supplemental Table S1C: GO terms predicted to be active by MGSA analysis. For comparison growth on straw compared to growth on malt extract, and growth on alder compared to malt extract

condition	GO ID	GO name	genes with this annotation in the genome	differentially expressed genes with this annotation	activation probability
alder-malt	GO:0016491	oxidoreductase activity	506	205	1
alder-malt	GO:0005975	carbohydrate metabolic process	226	72	1
alder-malt	GO:0071949	FAD binding	55	29	1
alder-malt	GO:0055085	transmembrane transport	513	165	1
alder-malt	GO:0055114	oxidation-reduction process	768	283	1
alder-malt	GO:0008152	metabolic process	387	158	1
alder-malt	GO:0008080	N-acetyltransferase activity	49	19	0.9998
alder-malt	GO:0006508	proteolysis	132	39	0.7976
straw-malt	GO:0008080	N-acetyltransferase activity	49	22	1
straw-malt	GO:0016491	oxidoreductase activity	506	192	1
straw-malt	GO:0005975	carbohydrate metabolic process	226	117	1
straw-malt	GO:0003824	catalytic activity	610	198	1
straw-malt	GO:0016787	hydrolase activity	165	54	1
straw-malt	GO:0071949	FAD binding	55	31	1
straw-malt	GO:0006508	proteolysis	132	53	1
straw-malt	GO:0055085	transmembrane transport	513	188	1
straw-malt	GO:0055114	oxidation-reduction process	768	279	1
straw-malt	GO:0000981	RNA polymerase II transcription factor activity, sequence-specific DNA binding	217	66	0.9982
straw-malt	GO:0042254	ribosome biogenesis	15	9	0.634

Supplemental Table S2

condition	CAZy Family	activity	genes with this annotation in the genome	differentially expressed genes with this annotation	activation probability
alder-straw	AA9	AA9 (formerly GH61) proteins are copper-dependent lytic polysaccharide monooxygenases (LPMOs); cleavage of cellulose chains with oxidation of various carbons (C-1, C-4 and C-6) has been reported several times in the literature;	49	35	1
alder-straw	CE1	acetyl xylan esterase; cinnamoyl esterase; feruloyl esterase; carboxylesterase; S-formylglutathione hydrolase; diacylglycerol O-acyltransferase; trehalose 6-O-mycolyltransferase	10	9	0.9826
alder-straw	GH11	endo-β-1,4-xylanase; endo-β-1,3-xylanase	6	6	0.935
alder-straw	GH7	endo-β-1,4-glucanase; reducing end-acting cellobiohydrolase; chitosanase; endo-β-1,3-1,4-glucanase	7	6	0.8518
alder-straw	GH131	broad specificity exo-β-1,3/1,6-glucanase with endo-β-1,4-glucanase activity;	4	4	0.7446
alder-straw	GH10	endo-1,4-β-xylanase; endo-1,3-β-xylanase; tomatinase; xylan endotransglycosylase	4	4	0.7428
alder-straw	GH5_5	endo-β-1,4-glucanase / cellulase; endo-β-1,4-xylanase; β-glucosidase; β-mannosidase; β-glucosylceramidase; glucan β-1,3-glucosidase; licheninase; exo-β-1,4-glucanase / cellodextrinase; glucan endo-1,6-β-glucosidase; mannan endo-β-1,4-mannosidase; cellulose β-1,4-cellobiosidase; steryl β-glucosidase; endoglycoceramidase; chitosanase; β-primeverosidase; xyloglucan-specific endo-β-1,4-glucanase; endo-β-1,6-galactanase; hesperidin 6-O-α-L-rhamnosyl-β-glucosidase; β-1,3-mannanase; arabinoxylan-specific endo-β-1,4-xylanase; mannan	5	5	0.6984

		transglycosylase			
solid-liquidExp	GH7	endo-β-1,4-glucanase; reducing end-acting cellobiohydrolase; chitosanase; endo-β-1,3-1,4-glucanase	7	7	0.937
solid-liquidExp	AA9	AA9 (formerly GH61) proteins are copper-dependent lytic polysaccharide monooxygenases (LPMOs); cleavage of cellulose chains with oxidation of various carbons (C-1, C-4 and C-6) has been reported several times in the literature;	49	26	0.813
solid-liquidExp	GH72	β-1,3-glucanosyltransglycosylase	7	6	0.7628
solid-liquidExp	AA7	glucooligosaccharide oxidase; chitooligosaccharide oxidase	25	14	0.6684
solid-liquidExp	GH3	β-glucosidase; xylan 1,4-β-xylosidase; β-glucosylceramidase; β-N-acetylhexosaminidase; α-L-arabinofuranosidase; glucan 1,3-β-glucosidase; glucan 1,4-β-glucosidase; isoprimeverose-producing oligoxyloglucan hydrolase; coniferin β-glucosidase; exo-1,3-1,4-glucanase; β-N-acetylglucosaminide phosphorylases	19	11	0.6038
solid-liquidSta	AA9	AA9 (formerly GH61) proteins are copper-dependent lytic polysaccharide monooxygenases (LPMOs); cleavage of cellulose chains with oxidation of various carbons (C-1, C-4 and C-6) has been reported several times in the literature;	49	33	1
solid-liquidSta	GH7	endo-β-1,4-glucanase; reducing end-acting cellobiohydrolase; chitosanase; endo-β-1,3-1,4-glucanase	7	7	0.9634
solid-liquidSta	CE16	acetylesterase active on various carbohydrate acetyl esters	5	5	0.847
solid-liquidSta	AA12	The pyrroloquinoline quinone-dependent oxidoreductase activity was demonstrated for the	6	5	0.745

		CC1G_09525 protein of Coprinopsis cinerea.			
solid-liquidSta	GH55	exo-β-1,3-glucanase; endo-β-1,3-glucanase	4	4	0.7364
solid-liquidSta	GH11	endo-β-1,4-xylanase; endo-β-1,3-xylanase	6	5	0.7024
stat-exp	CE8	pectin methylesterase	6	5	0.6276
straw-malt	AA9	AA9 (formerly GH61) proteins are copper-dependent lytic polysaccharide monooxygenases (LPMOs); cleavage of cellulose chains with oxidation of various carbons (C-1, C-4 and C-6) has been reported several times in the literature;	49	35	1
straw-malt	CE1	acetyl xylan esterase; cinnamoyl esterase; feruloyl esterase; carboxylesterase; S-formylglutathione hydrolase; diacylglycerol O-acyltransferase; trehalose 6-O-mycolyltransferase	10	9	0.9882
straw-malt	GH11	endo-β-1,4-xylanase; endo-β-1,3-xylanase	6	6	0.96
straw-malt	GH7	endo-β-1,4-glucanase; reducing end-acting cellobiohydrolase; chitosanase; endo-β-1,3-1,4-glucanase	7	6	0.877
straw-malt	GH10	endo-1,4-β-xylanase; endo-1,3-β-xylanase; tomatinase; xylan endotransglycosylase	4	4	0.7582
straw-malt	GH5_5	endo-β-1,4-glucanase / cellulase; endo-β-1,4-xylanase; β-glucosidase; β-mannosidase; β-glucosylceramidase; glucan β-1,3-glucosidase; licheninase; exo-β-1,4-glucanase / cellodextrinase; glucan endo-1,6-β-glucosidase; mannan endo-β-1,4-mannosidase; cellulose β-1,4-cellobiosidase; steryl β-glucosidase; endoglycoceramidase; chitosanase; β-primeverosidase; xyloglucan-specific endo-β-1,4-glucanase; endo-β-1,6-galactanase; hesperidin 6-O-α-L-rhamnosyl-β-glucosidase; β-1,3-mannanase; arabinoxylan-specific endo-β-1,4-xylanase; mannan	5	5	0.6948

		transglycosylase			

Supplemental Table S3

condition	KEGG pathway ID	KEGG pathway name	genes with this annotation in the genome	differentially expressed genes with this annotation	activation probability
alder-malt	ko00040	Pentose and glucuronate interconversions	35	19	1
alder-malt	ko01120	Microbial metabolism in diverse environments	246	92	1
alder-malt	ko04146	Peroxisome	54	35	1
alder-malt	ko00280	Valine, leucine and isoleucine degradation	49	26	1
alder-malt	ko00640	Propanoate metabolism	28	12	0.9992
alder-malt	ko00460	Cyanoamino acid metabolism	26	13	0.9868
alder-malt	ko00906	Carotenoid biosynthesis	4	4	0.9804
alder-malt	ko04978	Mineral absorption	7	4	0.9438
alder-malt	ko00052	Galactose metabolism	30	12	0.7968
alder-malt	ko04920	Adipocytokine signaling pathway	9	4	0.6656
alder-malt	ko04260	Cardiac muscle contraction	12	4	0.6062
alder-straw	ko00500	Starch and sucrose metabolism	65	32	1
alder-straw	ko01120	Microbial metabolism in diverse environments	246	96	1
alder-straw	ko04146	Peroxisome	54	33	1
alder-straw	ko00040	Pentose and glucuronate interconversions	35	16	0.9972

alder-straw	ko00190	Oxidative phosphorylation	73	25	0.9796
alder-straw	ko00520	Amino sugar and nucleotide sugar metabolism	49	18	0.974
alder-straw	ko00770	Pantothenate and CoA biosynthesis	23	10	0.9574
alder-straw	ko04142	Lysosome	40	13	0.955
alder-straw	ko00330	Arginine and proline metabolism	38	17	0.9466
alder-straw	ko00906	Carotenoid biosynthesis	4	4	0.9202
alder-straw	ko00600	Sphingolipid metabolism	21	11	0.8762
alder-straw	ko00910	Nitrogen metabolism	21	6	0.8466
alder-straw	ko00780	Biotin metabolism	9	5	0.7978
alder-straw	ko00740	Riboflavin metabolism	11	5	0.7602
alder-straw	ko04978	Mineral absorption	7	4	0.727
alder-straw	ko00380	Tryptophan metabolism	40	21	0.702
alder-straw	ko05130	Pathogenic Escherichia coli infection	13	5	0.6742
alder-straw	ko00640	Propanoate metabolism	28	13	0.6634
solid-liquidExp	ko00970	Aminoacyl-tRNA biosynthesis	40	28	0.9998
solid-liquidExp	ko01230	Biosynthesis of amino acids	116	58	0.9152
solid-liquidExp	ko00520	Amino sugar and nucleotide sugar metabolism	49	28	0.8352
solid-	ko03030	DNA replication	32	27	0.7924

liquidExp					
solid-liquidExp	ko03008	Ribosome biogenesis in eukaryotes	64	31	0.7612
solid-liquidExp	ko00280	Valine, leucine and isoleucine degradation	49	26	0.7048
solid-liquidExp	ko04111	Cell cycle - yeast	75	43	0.6586
solid-liquidExp	ko01524	Platinum drug resistance	29	18	0.6358
solid-liquidExp	ko03050	Proteasome	35	18	0.6236
solid-liquidSta	ko03008	Ribosome biogenesis in eukaryotes	64	38	1
solid-liquidSta	ko00500	Starch and sucrose metabolism	65	38	0.997
solid-liquidSta	ko00040	Pentose and glucuronate interconversions	35	25	0.9964
solid-liquidSta	ko03030	DNA replication	32	22	0.983
solid-liquidSta	ko00970	Aminoacyl-tRNA biosynthesis	40	22	0.9042
solid-liquidSta	ko00052	Galactose metabolism	30	20	0.882
solid-liquidSta	ko00564	Glycerophospholipid metabolism	39	22	0.8494
solid-	ko00770	Pantothenate and CoA biosynthesis	23	15	0.7834

liquidSta					
solid-liquidSta	ko00965	Betalain biosynthesis	17	12	0.6102
stat-exp	ko00040	Pentose and glucuronate interconversions	35	21	1
stat-exp	ko03010	Ribosome	99	29	1
stat-exp	ko01120	Microbial metabolism in diverse environments	246	60	0.9772
stat-exp	ko00280	Valine, leucine and isoleucine degradation	49	17	0.9676
stat-exp	ko00520	Amino sugar and nucleotide sugar metabolism	49	12	0.8906
stat-exp	ko00620	Pyruvate metabolism	41	11	0.8554
stat-exp	ko00260	Glycine, serine and threonine metabolism	47	11	0.845
stat-exp	ko00250	Alanine, aspartate and glutamate metabolism	31	9	0.7604
stat-exp	ko01110	Biosynthesis of secondary metabolites	355	94	0.7382
straw-malt	ko00500	Starch and sucrose metabolism	65	31	1
straw-malt	ko00040	Pentose and glucuronate interconversions	35	19	1
straw-malt	ko03008	Ribosome biogenesis in eukaryotes	64	23	1
straw-malt	ko00330	Arginine and proline metabolism	38	18	0.9978
straw-malt	ko00520	Amino sugar and nucleotide sugar metabolism	49	17	0.967
straw-malt	ko00980	Metabolism of xenobiotics by cytochrome P450	28	14	0.9512
straw-malt	ko00630	Glyoxylate and dicarboxylate metabolism	40	21	0.9136
straw-malt	ko00920	Sulfur metabolism	17	8	0.9056

straw-malt	ko00052	Galactose metabolism	30	15	0.897
straw-malt	ko00350	Tyrosine metabolism	50	20	0.838
straw-malt	ko00770	Pantothenate and CoA biosynthesis	23	11	0.7994
straw-malt	ko00910	Nitrogen metabolism	21	9	0.6518
straw-malt	ko01220	Degradation of aromatic compounds	32	17	0.6144

Supplemental Table S4

condition	GO ID	GO name	genes with this annotation in the genome	differentially expressed genes with this annotation	activation probability
alder-malt	GO:0016491	oxidoreductase activity	506	205	1
alder-malt	GO:0005975	carbohydrate metabolic process	226	72	1
alder-malt	GO:0071949	FAD binding	55	29	1
alder-malt	GO:0055085	transmembrane transport	513	165	1
alder-malt	GO:0055114	oxidation-reduction process	768	283	1
alder-malt	GO:0008152	metabolic process	387	158	1
alder-malt	GO:0008080	N-acetyltransferase activity	49	19	0.9998
alder-malt	GO:0006508	proteolysis	132	39	0.7976
alder-straw	GO:0016491	oxidoreductase activity	506	211	1
alder-straw	GO:0003824	catalytic activity	610	212	1
alder-straw	GO:0016787	hydrolase activity	165	57	1

alder-straw	GO:0071949	FAD binding	55	28	1
alder-straw	GO:0006508	proteolysis	132	53	1
alder-straw	GO:0055114	oxidation-reduction process	768	301	1
alder-straw	GO:0055085	transmembrane transport	513	176	0.9952
alder-straw	GO:0005975	carbohydrate metabolic process	226	110	0.988
solid-liquidExp	GO:0005524	ATP binding	497	231	1
solid-liquidExp	GO:0005515	protein binding	749	314	1
solid-liquidExp	GO:0003824	catalytic activity	610	256	1
solid-liquidExp	GO:0055085	transmembrane transport	513	230	1
solid-liquidExp	GO:0055114	oxidation-reduction process	768	341	0.9992
solid-liquidExp	GO:0006508	proteolysis	132	68	0.9848
solid-liquidExp	GO:0005975	carbohydrate metabolic process	226	115	0.9762
solid-liquidExp	GO:0005634	nucleus	389	180	0.9716
solid-liquidExp	GO:0016787	hydrolase activity	165	77	0.913
solid-liquidExp	GO:0016491	oxidoreductase activity	506	231	0.8836

solid-liquidExp	GO:0003676	nucleic acid binding	294	130	0.6472
solid-liquidSta	GO:0003824	catalytic activity	610	283	1
solid-liquidSta	GO:0005975	carbohydrate metabolic process	226	125	0.9898
solid-liquidSta	GO:0055114	oxidation-reduction process	768	335	0.9868
solid-liquidSta	GO:0008152	metabolic process	387	180	0.9798
solid-liquidSta	GO:0055085	transmembrane transport	513	264	0.975
solid-liquidSta	GO:0006508	proteolysis	132	66	0.8306
stat-exp	GO:0003824	catalytic activity	610	164	1
stat-exp	GO:0055114	oxidation-reduction process	768	232	1
stat-exp	GO:0016491	oxidoreductase activity	506	153	0.9988
stat-exp	GO:0071949	FAD binding	55	21	0.9874
stat-exp	GO:0005975	carbohydrate metabolic process	226	67	0.9518
stat-exp	GO:0055085	transmembrane transport	513	113	0.8948
straw-malt	GO:0008080	N-acetyltransferase activity	49	22	1
straw-malt	GO:0016491	oxidoreductase activity	506	192	1
straw-malt	GO:0005975	carbohydrate metabolic process	226	117	1

straw-malt	GO:0003824	catalytic activity	610	198	1
straw-malt	GO:0016787	hydrolase activity	165	54	1
straw-malt	GO:0071949	FAD binding	55	31	1
straw-malt	GO:0006508	proteolysis	132	53	1
straw-malt	GO:0055085	transmembrane transport	513	188	1
straw-malt	GO:0055114	oxidation-reduction process	768	279	1
straw-malt	GO:0000981	RNA polymerase II transcription factor activity, sequence-specific DNA binding	217	66	0.9982
straw-malt	GO:0042254	ribosome biogenesis	15	9	0.634
